# Phytochemicals Analysis and Medicinal Potentials of Hydroalcoholic Extract from *Curtisia dentata* (Burm.f) C.A. Sm Stem Bark

**DOI:** 10.3390/ijms13056189

**Published:** 2012-05-21

**Authors:** Sunday Oyewole Oyedemi, Blessing Ogochukwuamaka Oyedemi, Sunday Arowosegbe, Anthony Jide Afolayan

**Affiliations:** 1 School of Biological Sciences, University of Fort Hare, Alice 5700, South Africa; E-Mails: silvanusdemi@gmail.com (S.O.O.); sundayarowo@yahoo.com (S.A.); 2 School of Pharmacy, University of London, Brunswick Square, London WC1N 1AX, UK; E-Mail: blessing.mbabie@gmail.com

**Keywords:** *Curtisia dentata*, phytochemicals, antioxidant, cytotoxicity, antibacterial

## Abstract

*Curtisia dentata* (CD) is a vulnerable medicinal plant used for the treatment of stomach ailments in South Africa. However, there is a lack of sufficient data on its phytochemical components and medicinal properties. The phytochemical analysis of the extract was estimated using standard assay methods while its antibacterial activity was determined by the agar dilution method against selected bacteria. The antioxidant activity of the extract was done using ferric reducing power, 1,1-diphenyl-2-picrylhydrazyl **(**DPPH), 2,2′-azino-bis-3-ethylbenzothiazoline-6-sulfonic-acid (ABTS), nitric oxide (NO), hydrogen peroxide (H_2_O_2_) and lipid peroxidation (LPO). The cytotoxicity assay of the extract was assessed using the brine shrimp lethality test with LC_50_ value of 0.302 mg/mL. The antibacterial activity of the extract demonstrated an appreciable broad spectrum activity against the tested bacteria with minimum inhibitory concentration (MIC) ranges between 5000 and 0.5 mg/L. Both phenol and flavonoid concentrations were 14.86 mg tannic acid equivalent/g and 13.64 mg quercetin equivalent/g, respectively. The percentage composition of saponins (13.26) was highest, followed by steroids (1.42), while alkaloids and tannins had the same value of 0.51. Similarly, IC_50_ values of the extract against DPPH, ABTS, H_2_O_2_, LPO and NO were 0.017, 0.018, 0.159, 0.06 and 0.052 mg/mL, respectively. The reducing power of the extract was found to be concentration dependent. Our data suggest that the 70% ethanol extract from the CD extract has antibacterial and antioxidant properties due to the presence of bio-active compounds and thus support its folkloric use in the treatment of diseases.

## 1. Introduction

The widespread nature of antimicrobial resistance to current antibiotics has posed a serious threat to global public health [1]. It has been shown that about 45 percent of isolates from patients in South Africa are resistant to treatment with penicillin, erythromycin, ampicillin, clindamycin, tetracycline and sulphonamides due to the quick adaptation of bacteria to new environmental conditions [2]. The exposure of organisms to both exogenous and endogenous factors has been reported to generate a wide range of reactive oxygen species, resulting in hundreds of disorders in humans [3]. To combat these challenges, researchers have discovered the use of medicinal plants as a strategy for the production of effective novel compounds to retard or alleviate the extent of oxidative deterioration [4]. Medicinal plants remain the nature reservatory of biological active compounds with biochemical and therapeutic properties. These properties include antimicrobial and antioxidant compounds that are increasingly harnessed for human benefit [5]. The majority of people, especially those living in rural communities, depend heavily on folklore medicine for their primary healthcare owing to their ready availability, relative safety and affordability over modern medicine [5]. However, some of these plants have not been investigated for their medicinal purposes. In addition, the increasing use of plant extracts in food, cosmetics and pharmaceutical industries demands toxicity assessments to justify their continued therapeutic application.

*Curtisia dentata* (Burm.f.) C.A. Sm, locally named Assegaai (Afrikaans) and umLahleni (Xhosa, Zulu) in South Africa, belongs to the Cornaceae family. The plant is an attractive medium-sized tree with a dark bark that is fissured in square patches. *C. dentata* (CD) leaves are smooth, glossy and ovate, broadly elliptic and up to 10 cm in length [6]. The flowers are small, with rounded to oval fleshy bitter berries that are about 10 mm in diameter, are white and turn red when ripe [7]. Its distribution is mostly on the mountains, in the evergreen forests and along the margins of forests and grasslands of southern Africa. This plant is commonly found in the forests of Limpopo, KwaZulu-Natal, and the eastern and western Cape Provinces of South Africa [6]. It is classified as vulnerable, declining, conservation-dependent in KwaZulu-Natal where the bark is traded annually as a special herbal mixture for the treatment of disease [7]. In southern Africa, the stem bark of CD is used traditionally for the management of stomach ailments, diarrhea and as a blood purifier as well as an aphrodisiac [7]. It is also used in the eastern Cape of South Africa for the treatment of heart-water disease in cattle [8]. The plant was discovered recently as an alternative remedy for the management of obesity in the South Africa traditional medicine [9]. Previous work reported the antifungal activity of the aqueous, acetone and ethanol extract of the leaves, twigs and stem bark of CD [10,11]. The folklore medicine has been reported to act against some parasites and nematodes [6]. Recent studies by Doughari *et al*. [12] revealed the presence of some phytochemicals in various parts of the plant but this was not quantified. In addition, some compounds such as betulinic acid, ursolic acid and 2-alpha hydroxyursolic acid with antimicrobial properties have been isolated from CD [6]. Different solvent systems of the extract have been tested against some organisms but there have been no reports in the scientific literature on the hydroalcoholic extracts considering the herbal preparation of this plant among users.

Therefore the present study was conducted to provide scientific data on the antibacterial and antioxidant properties of hydroalcoholic extracts from the CD stem bark. In addition, quantified phytochemical analysis and brine shrimp lethality test for preliminary assessment of toxicity was performed.

## 2. Results and Discussion

### 2.1. Phytochemical Components

The quantitative phytochemical analysis of the hydroalcoholic extract from the *Curtisia dentata* stem bark is presented in Table 1. Our data showed a high percentage composition of saponins (13.26 ± 0.01) followed by steroids (1.42 ± 0.01), while alkaloids and tannins had the same concentration of 0.51 ± 0.02. The results of total phenolic contents of the extract was found to be 14.86 mg of tannic acid equivalent expressed from the standard tannic acid curve: *Y* = 0.1216*x*, *R*^2^ = 0.93651. Total flavonoid was found to be 13.64 mg of the quercetin equivalent extrapolated from the standard quercetin curve: *Y* = 0.0255*x*, *R*^2^ = 0.9812. These phytochemicals are well known to perform different biological activities in medicinal plants including antimicrobial and antioxidant properties through various mechanisms of action.

The presence of tannins in CD extracts lend credence to the ethnomedicinal use of the extract in the treatment of various diseases. Several researchers have reported the importance of tannins for the prevention and treatment of cancer, treatment of ulcerated tissues, inhibition of lipid oxidation, antibacterial and amelioration of renal failure [13,14]. Hence, the amount of this compound in the CD extract could support the antibacterial and antioxidant potential observed in this study. An alkaloid is one of the largest secondary metabolites in plants with potential to protect the cells against foreign organisms due to its toxic nature. This nature is responsible for the medicinal values of various plants used for the management of human cancer, anti-malaria, analgesic, antiseptic and bactericidal activities [15]. The presence of alkaloids in CD extract could be a useful source to support the ethnomedicinal uses of this folklore medicine in the South Africa traditional system of medicine as herbal remedies. Saponins, responsible for most biological activities related to cell growth and division in humans [16], were also present in CD extract and thus may be a potential source of anticancer agents. Steroidal compounds are of importance and play a vital role as antibacterial, antiviral and aphrodisiac agents [17]. The presence of steroids in the extract could support the antibacterial properties observed in this study and its usefulness in the treatment of sexual dysfunction. It is not unlikely that these bioactive compounds found in *Curtisia dentata* are the reason for the antibacterial and antioxidant activities exhibited by CD.

Polyphenolic compounds such as phenols and flavonoids are important plant components with significant antioxidant activity and a wide range of biological activities including antibacterial, anti-inflammatory, analgesic and anti-allergic properties [18]. These compounds have been indicated in several studies as free radicals scavengers against superoxide anion, lipid peroxyl and hydroxyl radicals and so highlights many of their health-promoting functions in organisms, which is important for prevention of diseases associated with oxidative damage of membranes, proteins and DNA [5].

### 2.2. Ferric Reducing Power Activity

**Figure 1:**
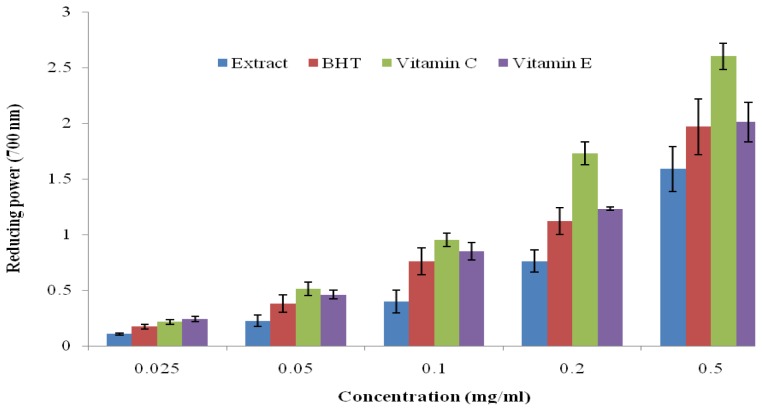
Reducing power of hydroalcoholic extract of *C. dentata*, BHT, vitamin C and vitamin E by spectrophotometric detection of Fe^3+^ to Fe^2+^ transformation. Results are mean ± SD of triplicate samples.

The reducing power of many plant extracts has been used to correlate their antioxidant potential [5]. The antioxidant activity was determined based on the ability of the extract to transform Fe^3+^ to Fe^2+^ in a redox linked colorimetric reaction that involves an electron transfer [19]. Iron (II) was then monitored by measuring the formation of Perl’s Prussian blue at 700 nm. The extracts showed a good reducing power in a concentration dependent manner but significantly lower when compared with the standard drugs in this order; Vitamin E > Vitamin C > BHT > extract (Figure 1). The antioxidative effects of the CD extract as shown in this study could be attributed to the presence of phenolic compounds with potential to donate electrons and to terminate radical chain reactions. A similar trend was observed by Oyedemi *et al*. [5] in regard to the antioxidant property of hydroalcoholic extract from *Scotia latifolia* stem bark.

### 2.3. DPPH Radical Scavenging Activity

The *in vitro* scavenging potential of the plant extract against DPPH stable nitrogen centered radical was determined by measuring the changes in absorbance at 517 nm. The resulting decolorization of purple color to yellow is stoichiometric with respect to the number of electrons captured based on the concentration of the extract. The IC_50_ values for the extract, BHT and rutin were 0.017, 0.024 and 0.019 mg/mL respectively (Table 2). Our results showed that the extract had a good DPPH scavenging property when compared with the reference drugs. A similar result was reported by Tural and Koca [20] in regard to the free radical scavenging properties of *Corneus mas*, which belongs to the same family as *C. dentata*. Therefore, the data obtained from this study justified the ethnomedicinal use of this plant in the treatment of pathological disease emanating from oxidative stress.

### 2.4. ABTS Radical Scavenging Activity

ABTS is a compound frequently used in phytomedicine research to measure the antioxidant properties of plants for better elucidation of their biological properties. The radical is generated through the oxidation of ABTS to an intensely colored nitrogen centered cation by reacting with potassium persulfate for 12–14 h. In this study, the extract at various concentrations (0.0078 to 0.5 mg/mL) scavenged the radical in a concentration dependent manner. The IC_50_ values obtained for the extract (0.018 mg/mL) showed a comparable activity with the standard BHT (0.015 mg/mL) and rutin (0.016 mg/mL), which are derivatives of phenolic compounds (Table 2). Our observations agreed with several scientific reports but contradicted that of Wang *et al*. [21] who found that some compounds which inhibited DPPH* did not show ABTS* scavenging property. The present study demonstrated the capability of the plant extract to scavenge both radicals in different systems and thus suggest its usefulness as a therapeutic agent for the treatment of pathological diseases related to free radicals.

### 2.5. Nitric Oxide Scavenging Activity

Nitric oxide is an important cellular signaling molecule needed in low amounts to protect some vital organs in the human body, especially the liver and heart, from ischemic damage and blood pressure, respectively. The protective mechanism could be linked to the activation of the transcription factor in the iNOS gene expression as a response to inflammation [22]. However, the over production of this chemical has been implicated in the pathogenesis of various physiological and pathological disorders such as carcinomas, juvenile diabetes, ulcerative colitis and arthritis [22]. In this study, NO* was generated from sodium nitroprusside in aqueous solution and it reacted with oxygen to form a nitrite and later peroxynitrite when combined with superoxide. The result of the scavenging capability of the CD extract against nitric oxide radicals is shown in Table 2. The IC_50_ value for the extract, rutin and BHT were 0.052, 0.018 and 0.017 mg/mL, respectively. Our data demonstrated the extract as a potent scavenger of nitric oxide and as a result could be useful in the management of inflammatory reactions that are detrimental to human health.

### 2.6. Hydrogen Peroxide Scavenging Capacity

Hydrogen peroxide is an important reactive oxygen species (ROS) due to its ability to penetrate biological molecules. It is derived from a superoxide radical by the action of superoxide dismutase and is further reduced to a hydroxyl radical (OH*) by the enzymatic action of glutathione peroxidase and catalase in the presence of iron or copper. The OH* generated is an extremely reactive radical capable of damaging macromolecules such as membrane lipids, proteins and carbohydrates in the biological system [5]. It has been reported to induce breakage in a DNA strand and chemical changes in the deoxyribose and nitrogenous base [5]. Consequently, for a hydroxyl radical to be reduced in the body, there is a need for the excess hydrogen peroxide generated to be scavenged. The scavenging potential of the CD extract against hydrogen peroxide is presented in Table 2 using BHT and gallic acid as positive controls. *Curtisia dentata* extract had strong potential to eliminate hydrogen peroxide which is depicted with the IC_50_ value of 0.159 mg/mL but this is significantly low (*p* > 0.05) when compared with BHT (0.096 mg/mL) and rutin (0.047 mg/mL). The present result showed that the CD extract has significant effect in neutralizing the harmful effect of hydrogen peroxide.

### 2.7. Lipid Peroxidation

Lipid peroxidation is one of the major outcomes of free radical-mediated injury to tissue. The oxidation of lipids alters the physicochemical properties of membranes, resulting in severe cellular dysfunction [23]. Our study investigated the inhibitory potential of the *C. dentata* extract against lipid peroxidation using egg-yolk homogenates as lipid-rich media. The results obtained from this study showed the scavenging capacity of the extract, BHT and Gallic acid against lipid peroxidation with IC_50_ values of 0.06, 0.102 and 0.215 mg/mL respectively (Table 2). The plant extract showed a capacity to deplete lipid peroxidation that is higher than that of standard gallic acid and BHT. It can be inferred from this study that the CD extract has the potential to play a crucial role in protecting the physicochemical properties of membrane bilayers from free radical-induced cellular dysfunction which could be adduced to the synergistic effect of the antioxidant compounds present in this plant.

### 2.8. Antibacterial Activity

The antibacterial activity of the 70% CD stem bark was tested against ten Gram positive and five Gram negative bacteria using agar dilution method. The MIC results summarized in Table 3 show that the crude extract had broad spectrum activity and was able to prevent the growth of the tested bacteria between the range of 5000 and 19.5 mg/L. According to the classification of Rios and Rico [24] the crude extract of CD could be considered to possess significant (MIC < 100 mg/L), moderate (100 < MIC = 625 mg/L) or weak (MIC > 625 mg/L) activity against the corresponding pathogens. The CD extract was found inactive against *B. pumilus* and *E. coli*. A significant antibacterial activity was observed against *E. faecalis*, *P. vulgaris*, *S. mercescens*, *S. aurues* OKI and OK3 with MIC value of 19.5 mg/L but had moderate activity against *B. cereus*, *P. aeruginosa* and *K. pnuemoniae* with MIC range from 156.2 to 312.5 mg/L.

The crude extract showed moderate antibacterial activity against multidrug-resistant (MDR) *S. aureus* strains with MIC values ranging from 256 to 512 μg/mL. SA-1199B is a multidrug-resistant strain that overexpresses the NorA efflux mechanisms with a high level of resistance to certain fluroquinolones. EMRSA-15 and EMRSA-16 are major epidemic methicillin-resistant *S. aureus* strains in the UK Hospitals [25,26] and ATCC 25923 a standard laboratory strain. Norfloxacin was more active against these strains of bacteria than the crude extract with MIC of 0.5 to 128 μg/mL. However, being a natural product, the extract has been reported to be much safer than the antibiotics. The activity displayed by this extract against MDR strains suggests that CD extract may not be a substrate for the most resistance mechanisms to current antibacterial agents, considering the crude nature of the solvent extract used in this study. Early observation by McGaw *et al*. [9] reported antibacterial activity of ethanol and aqueous stem bark extracts of the plant against *Bacillus subtilis* which was also confirmed in this study using the 70% ethanol extract. Our findings are comparable to data reported by Shai *et al*. [10] who demonstrated a moderate inibitory effect of acetone stem bark extracts of the plant on *Pseudomonas aeruginosa* using microdilution method but contradict the MIC values obtained for *E. coli*, *E. faecalis* and *S. aureus*. The data obtained for *Proteus vulgaris* concurred with the report of Dulger and Gonuz [27] on ethanol extracts of *Comus mas* (a member of the same family). The observed antibacterial activity against some bacteria could be explained by the presence of potentially active classes of secondary metabolites with a strong ability to penetrate or disrupt the multilayered structure of Gram negative bacteria (Table 1). Compounds such as flavonoids, polyphenols, tannins, and alkaloids have been reported to possess antimicrobial properties and thus could be responsible for this observation [5]. Our observation strongly suggests that *Curtisia dentata* can be an effective herbal remedy in the treatment of infections caused by these pathogens.

### 2.9. Brine Shrimp Lethality Test

**Figure 2:**
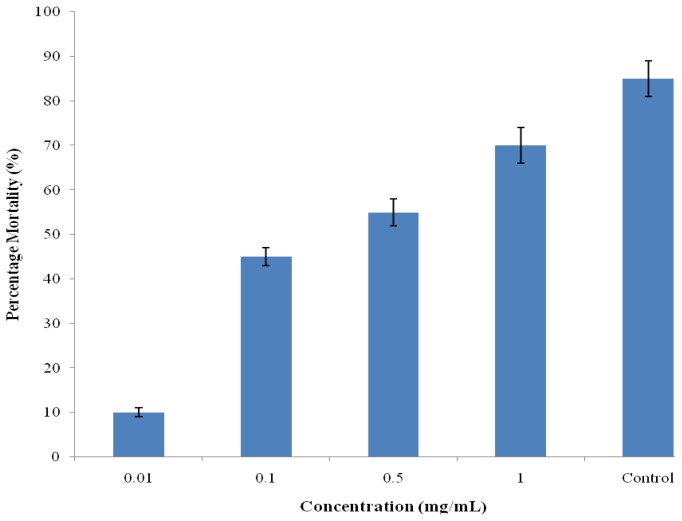
Brine shrimp cytotoxicity of hydroalcoholic stem bark extract of *Curtisia dentate*.

The brine shrimp lethality test is considered a convenient tool for the preliminary assessment of toxicity and the detection of fungal toxins in the test materials [28]. A number of novel antitumour and pesticidal natural products have been isolated using this bio-assay. The extracts had no toxic effect against brine shrimp with LC_50_ values of 0.302 mg/mL compared to the reported standard cyclophosphamide [29] and vincristine sulfate [30] with LC_50_ values of 16.3 and 0.52 μg/mL respectively. Cytotoxicity test of betunilic acid and lupeol isolated from CD extract as reported by Shai *et al*. [6] indicated toxic effect using Vero cells but showed otherwise using crude extract on brine shrimps assay (Figure 2). Although, the plant extracts were more active against tested bacteria, they exhibited very low toxicity against brine shrimps. This observation may suggest inherent selectivity of the extracts; hence, supporting its ethnomedicinal use for the treatment of bacterial infections.

## 3. Material and Methods

### 3.1. Plant Collection and Extract Preparation

The stem bark of the CD was collected from the Amathole Mountain in the Eastern Cape Province of South Africa in April 2010. The plant was identified and authenticated by DS Grierson of the Department of Botany, University of Fort Hare. A voucher specimen (SUNMED) was deposited at the Giffen herbarium of the University. The bark was oven–dried at 50 °C for 14 days, pulverized and stored in an airtight container for further use. The powdered plant material (100 g) was extracted in 1 L of 70% *v*/*v* (alcohol:water) on a mechanical shaker (Labotec Scientific Orbital Shaker, SA) for 48 h. The extract was filtered using a Buchner funnel and Whatman No.1 filter paper. The filtrate was concentrated under reduced pressure at 40 °C to recover ethanol and was later air-dried in the fume hood to dryness. The yield after extraction was 14.28 g, which was later reconstituted in distilled water to give the desired concentrations needed for this study.

### 3.2. Bacterial Strains

Bacteria isolates used in this study are reference strains obtained from the Applied and Environmental Microbiology Research Group (AEMREG), Department of Biochemistry and Microbiology, University of Fort Hare, Alice, South Africa. These bacteria were chosen for their pathological effects on humans and in the deterioration of food products. These included Gram^+^ bacteria: *Bacillus cereus* (10702), *Bacillus pumilus* (ATCC 14884), *Enterobacter faecalis* (KZN) and six Gram^−^ bacteria: *Proteus vulgaris* (KZN), *Serratia mercescens* ATCC (9986), *Acinectobacter calcaocenticus* (UP), *Pseudomonas aeruginosa* (ATCC 19582), *Escherichia coli* (ATCC 25922) and *Klebsiella pnuemoniae* (KZN). Also included were *Staphylococcus aureus* (OK1 and OK3) isolated from marines. Multidrug resistant *Staphylococcus aureus* SA-1199B and standard resistant strain ATCC 25923, EMRSA-15 and EMRSA-16 were obtained and tested in the Simon Gibbons’s Microbiology Tissue Unit, UCL School of Pharmacy. The inoculums of the test organisms were prepared using the colony suspension method [31]. The bacterial strains and isolates were grown at 37 °C overnight and maintained on nutrient agar and later standardized following the McFarland turbidity of 0.5 at 600 nm to achieve 5 × 10^5^ colony forming units per mL (CFU/mL).

### 3.3. Minimum Inhibitory Concentration (MIC) Determination of Bacteria

The minimum inhibitory concentrations (MICs) of the antibiotics and plant extracts were determined using the standard method of the Clinical Laboratory Standards Institute [32]. Dilutions of the antibiotics, ranging from 0.001–0.824 mg/mL in nutrient agar (Biolab), were prepared by incorporating the antibiotic stock solution into molten agar at 50 °C. Different concentrations of the extracts were prepared to final concentrations in the range of 5–0.05 mg/L. A volume of 1 mL from each dilution of the extract was mixed with 19 mL of molten sensitivity test agar (Biolab) at 50 °C and poured into sterile Petri dishes allowing the agar to set. The surface of the agar was allowed to dry before streaking with standardized overnight broth cultures of the test bacteria. Plates were incubated at 37 °C for 24 h under aerobic conditions. The MIC was defined as the lowest concentration of the antibiotic or extracts that completely inhibited visible growth of the test organism. The resistant strains of *S. aureus* were cultured on nutrient agar (Oxoid) and incubated for 24 h at 37 °C prior to MIC determination. A volume of 100 μL of sterile Mueller-Hinton broth (MHB: Oxoid) containing 20 mg/L and 10 mg/L of Ca^2+^ and Mg^2+^ respectively was dispensed into 10 wells of a 96 well microliter plate. The plant extract was dissolved in dimethylsulfoxide (DMSO) and further diluted in MHB to give a stock solution. A volume of 100 μL of the antibacterial agent stock solution (2.048 mg/L) was serially diluted into each well and then mix with 100 μL of bacterial inoculums to give a final concentration range from 512–1 mg/L. All procedures were performed in duplicate and the plates incubated for 18 h at 37 °C. 20 μL of a 5 mg/mL methanolic solution of 3-[4,5-dimethylthiazol-2-yl]-2,5-diphenyltetrazolium bromide (MTT; Sigma) was added to each well and incubated for 30min. A blue coloration indicated bacterial growth. The MIC was recorded as the lowest concentration at which no visible growth was observed.

### 3.4. Determination of Total Phenols

The amount of phenol in the CD extract was determined spectrophotometrically using the modified method of Zovko [33] with the Folin-Ciocalteu reagent. An aliquot of the extract (1 mg/mL) was mixed with 5 mL Folin-Ciocalteu reagent (previously diluted with water 1:10 *v*/*v*) and 4 mL (75 g/L) of sodium carbonate. The tubes were vortexed for 15 s and allowed to stand for 30 min at 40 °C for color development. Absorbance was then measured at 765 nm using the AJI-C03 UV-VIS spectrophotometer. Results were expressed as mg/g of tannic acid equivalent.

### 3.5. Determination of Flavonoids

The amount of flavonoids in the stem bark extracts was also determined by using the aluminum colorimetric assay method [5]. A volume of 0.5 mL of 2% AlCl_3_ ethanol solution was added to 0.5 mL of the sample solution. After 1 h at room temperature, the absorbance was measured at 420 nm. A yellow color indicated the presence of flavonoids. Extract samples were evaluated at a final concentration of 0.1 mg/mL. Total flavonoid contents were calculated as mg/g of quercetin.

### 3.6. Determination of Tannin Contents

The tannin content was determined according to the method of AOAC [34] with some modifications. A plant sample weighing 0.20 g was added to 20 mL of 50% methanol, vortexed vigorously and later incubated at 80 °C in a water bath for 1 h. The filtrate (0–10 ppm) of the sample after filtration was mixed with 20 mL of distilled water, 2.5 mL of Folin-Denis reagent and 10 mL of 17% aqueous Na_2_CO_3_. The mixture was made up to 100 mL with distilled water, mixed and allowed to stand for 20 min. A bluish-green color developed at the end of the reaction mixture. The absorbance of the tannic acid standard solutions and the sample was measured after color development at 760 nm. Results were expressed as mg/g of tannic acid equivalent using the calibration curve: *Y* = 0.0593 *x* − 0.0485, *R*^2^ = 0.9826, where *x* is the absorbance and *Y* is the tannic acid equivalent.

### 3.7. Determination of Alkaloids Contents

Total alkaloids contents were quantitatively determined according to the method of Harborne [35]. A volume of 200 μL of 10% acetic acid prepared in ethanol was added to 5 g of powdered plant sample, covered and allowed to stand for 4 h. The filtrate obtained after filtration of plant sample was reduced to one-fourth of its original volume over a water bath. Concentrated ammonium hydroxide was added drop-wisely to the extract, pending the completion of precipitation. The whole solution was allowed to settle and re-filtered after washing with dilute ammonium hydroxide. The residue obtained was dried, weighed and the percentage composition was determined using the formula: % alkaloid = final weight of the sample**/**initial weight of the extract × 100.

### 3.8. Determination of Saponins Content

Saponins content was determined using the method of Obadoni and Ochuko [36]. The plant sample (20 g) was added to 100 mL of 20% aqueous ethanol and kept in a shaker for 30 min. The samples were heated over a water bath for 4 h at 55 °C and then filtered to collect the residue which was later re-extracted with 200 mL of 20% aqueous ethanol. The extracts obtained were concentrated over a water bath at 90 °C to approximately 40 mL. The concentrate was transferred into a 250 mL separatory funnel and extracted twice with 20 mL diethyl ether. The ether layer was discarded while the aqueous layer was retained and to which 60 mL *n*-butanol was added. The *n*-butanol extracts were washed twice with 10 mL of 5% aqueous sodium chloride. After evaporation, the samples were dried in the oven at 40 °C to a constant weight. The saponins content was calculated using the formula: % saponin = final weight of sample/initial weight of extracts × 100.

### 3.9. Ferric Reducing Power

The reducing power of the CD extract was evaluated according to the method of Yen and Chen [37] with a little modification. A volume of 1.0 mL of different concentrations (0.025–0.5 mg/mL) of extract, BHT, ascorbic acid and alpha tocopherol (Vitamin E) prepared in distilled water was mixed in the reacting mixture containing 2.5 mL of 0.2 M phosphate buffer (pH 6.6) and 2.5 mL of K_3_Fe(CN)_6_ (1% *w*/*v*). The resulting mixture was incubated at 50 °C for 20 min, followed by the addition of 2.5 mL of TCA (10% *w*/*v*). After vigorous shaking, 2.5 mL of the solution was mixed with 2.5 mL of distilled water and 0.5 mL of FeCl_3_ (0.1%, *w*/*v*). The absorbance was measured at 700 nm against a blank sample (without extract).

### 3.10. DPPH Radical Scavenging Activity

The scavenging activity of the plant extract against DPPH^.+^ was done according to the method described by Liyana-Pathiranan and Shahidi [38]. One milliliter of 0.135 mM DPPH prepared in methanol was mixed with 1.0 mL of various concentrations (0.0078–0.5 mg/mL) of extract, BHT or rutin. The reaction mixture was vortexed thoroughly and left in the dark at room temperature for 30 min. The absorbance of the mixture was measured at 517 nm. The ability of the plant extract to scavenge DPPH^.+^ was calculated by the equation: DPPH^.+^ scavenging activity = {(Abs_control_ − Abs_sample_)}/(Abs_control_} × 100 where Abs_control_ is the absorbance of DPPH^.+^ + methanol; Abs_sample_ is the absorbance of DPPH radical + sample extract/standard.

### 3.11. ABTS Radical Scavenging Activity

The ability of the CD extract to scavenge ABTS^.+^ was assessed using the method of Re *et al*. [39]. The radical was generated by mixing two stock solutions of 7 mM ABTS and 2.4 mM potassium persulfate in the same ratio and allowing the solutions to react in the dark for 12 h at room temperature. The resulting solution was further diluted by methanol (1.0 mL of ABTS radical in 60 mL of methanol) to obtain an absorbance of 0.706 ± 0.001 units. Different concentrations (0.0078–0.5 mg/mL) of extract and standard drugs was allowed to react with the ABTS radical in the dark for 7 min and were later measured for absorbance at 734 nm. The percentage inhibition of ABTS^+^ by the extract or BHT or rutin was calculated and compared with the standard drugs using this equation. ABTS radical scavenging activity = {(Abs_control_ − Abs_sample_)}/(Abs_control_} × 100 where Abs_control_ is the absorbance of ABTS radical + methanol; Abs_sample_ is the absorbance of ABTS radical + sample extract/standard.

### 3.12. Nitric Oxide Scavenging Activity

The method of Ebrahimzadeh *et al*. [40] was adopted to determine the scavenging activity of *C. dentata* against the nitric oxide radical. A volume of 2 mL sodium nitroprusside (10 mM) was prepared in 0.5 mM phosphate buffer saline (pH 7.4) mixed with 0.5 mL of plant extract or BHT or rutin at various concentrations (0.025–0.5 mg/mL). The mixture was incubated at 25 °C for 150 min. After incubation, 0.5 mL of the solution was withdrawn and mixed with 0.5 mL of Griess reagent containing 1.0 mL of 0.33% sulfanilic acid reagent prepared in 20% glacial acetic acid at room temperature for 5 min with 1 mL of napthyethylenediamine dichloride (0.1% *w*/*v*)]. The mixture was furthered incubated at room temperature for 30 min followed by the measuring of the absorbance at 540 nm. The amount of nitric oxide radical inhibited by the extract was calculated using this equation: Nitric oxide radical scavenging activity = {(Abs_control_ − Abs_sample_)}/(Abs_control_} × 100 where Abs_control_ is the absorbance of Nitric oxide radical + methanol; Abs_sample_ is the absorbance of Nitric oxide radical + sample extract/standard.

### 3.13. Assessment of Lipid Peroxidation

The effect of the CD extract was evaluated on lipid peroxide formation with an egg yolk homogenate using a modified thiobarbituric acid-reactive species (TBARS) assay [23]. The egg homogenate (0.5 mL, 10% in distilled water, *v*/*v*) and 0.1 mL of the stem bark extract of CD was mixed in a test tube and made up to 1 mL with distilled water. A volume of 0.05 mL of 0.07 M FeSO_4_ was added to the above mixture and further incubated for 30 min, to induce lipid oxidation. Thereafter, 1.5 mL of 20% acetic acid (pH 3.5), 1.5 mL of 0.8 % *w*/*v* TBA prepared in 1.1 % *w*/*v* sodium dodecyl sulfate and 0.05 mL of 20 % *w*/*v* TCA were added, vortexed and then heated in a boiling water bath for 60 min. After cooling, 5.0 mL of 1-butanol was added to each tube and centrifuged at 3000 rpm for 10 min. The absorbance of the upper layer solution was measured at 532 nm. For the blank, 0.1 mL of distilled water was used instead of the extract.

### 3.14. Brine Shrimp Lethality

A brine shrimp (*Artemia salina*) lethality bioassay was carried out to determine the preliminary cytotoxicity effect of the hydroalcoholic stem bark extract of CD [28]. The eggs of the shrimp were purchased from an aquarium shop (East London, South Africa) and hatched in sea water for 24 h at 25 °C to mature shrimp called nauplii. The plant extract was tested at 5 to 0.625 mg/mL by dissolving 10 mg of the extract in 10% DMSO prepared in sea water. After incubation, 10 brine shrimp larvae were introduced into vials containing the plant extracts at various concentrations (0.001–1.0 mg/mL). After 24 h, the number of surviving shrimps at each concentration was counted using a magnifying glass. A vial containing 50 μL DMSO diluted to 5 mL was used as a control. The lethal concentration of extract resulting in 50% mortality (LC_50_) was extrapolated from the graph.

### 3.15. Statistical Analysis

Data were expressed as means ± SD (standard deviation) of three replicates and were statistically analyzed using one way analysis of variance (ANOVA). Means were separated by the Duncan multiple test using SAS. Values were considered significant at *p* < 0.05.

## 4. Conclusions

The present study revealed that 70% ethanol extract of *C. dentata* stem bark has selective antibacterial activity against tested bacteria without toxic effects. Strong antioxidant and free radical scavenging properties of CD extract suggested its use as a natural source of antioxidant and anti-aging factor to curtail progression of radical related diseases. The antibacterial activities of the extract against tested bacteria and MDR strains showed that it has potential to be used for the development of therapeutic agents for the treatment of infection caused by these bacteria. A further study to characterize the active principles and to elucidate the mechanisms of action of CD extract is needed to confirm its ethnomedicinal usage.

## Figures and Tables

**Table 1:** Phytochemical analysis of hydroalcoholic stem bark extracts of *C. dentata*.

Phytochemicals	Amount	R^2^	Regression equation
1 Total Phenols (mg/g)	14.86 ± 0.05	0.9365	*Y* = 0.1216*x*
2 Total Flavonoids (mg/g)	13.64 ± 0.03	0.9812	*Y* = 0.0255*x*
Saponins (%)	13.26 ± 0.01	-	-
Tannins (%)	0.51 ± 0.02	-	-
Alkaloids (%)	0.51 ± 0.11	-	-
Steroids (%)	1.42 ± 0.12	-	-

Data are expressed as means ± SD; *n* = 3; Values along a row with different superscripts are significantly different (*p* < 0.05). Mean with the same subscript in the same column are:

1 Expressed as mg tannic acid/g of dry plant materials

2 Expressed as mg quercetin/g of dry plant materials.

**Table 2:** Antioxidant and free radical scavenging activity of hydroalcoholic extract of *C. dentata* stem bark.

Free radical scavenging activity of *Curtisia dentata* (IC_50_ mg·mL^−1^)					

Sample	DPPH	ABTS	NO	H_2_O_2_	LPO
*Curtisia dentata*	0.017	0.018	0.052	0.159	0.06
Rutin	0.019	0.016	0.018	0.047	-
BHT	0.024	0.015	0.017	0.096	0.102
Gallic acid	-	-	-	-	0.215

Values are the mean of three replicates.

**Table 3:** Minimum Inhibitory Concentration (MIC) of *Curtisia dentata* stem bark.

Antibacterial activities of *C. dentata* extract				

Bacteria	Gram +/−	CD extract (mg/L)	Streptomycin (mg/L)	Norfloxacin (mg/L)
*Bacillus cereus* ATCC 10702	+	312.5	2	-
*Bacillus pumilus* ATCC 14880	+	5000	2	-
*Pseudomonas aeruginosa* ATCC 19582	−	312.5	8	-
*Serratia mercescens* ATCC 9986	−	19.5	4	-
*Acinectobacter calcaocenticus* UP	−	1250	2	-
*Klebsiella pneumoniae* KZN	+	156.2	2	-
*Proteus vulgaris* KZN	−	19.5	4	-
*Enterobacter faecalis* KZN	+	19.5	2	-
*Staphylococcus aureus* OK1	+	19.5	4	-
*Staphylococcus aureus* OK3	+	19.5	4	-
*Escherichia coli* ATCC 14884	−	5000	8	-
† *Staphylococcus aureus* 1199B	+	512	-	32
† *Staphylococcus aureus* ATCC 25923	+	256	-	1
† *EMRSA-* 15	+	512	-	0.5
† *EMRSA-* 16 (mecA)	+	512	-	128

All MICs were determined in duplicate.

† indicates resistant bacteria;

EMRSA: Extended methicilin resistant *Staphylococcus aureus*.

## References

[1] (2001). General Guidelines for Methodologies on Research and Evaluation of Traditional Medicine.

[2] Lesley M., Hui W., Avril W., Robin H., Minchun C., Keith P. (2001). Prevalence of serotypes and molecular epidemiology of streptococcus pneumoniae strains isolated from children in Beijing, China: Identification of two novel multiply-resistant clones. Microb. Drug Resist.

[3] Halliwell B. (1992). Reactive oxygen species in living systems: Source, biochemistry, and role in human disease. Am. J. Med.

[4] Walter H.L., Memory P.E. (1995). Medicinal plants as sources of New Therapeutics. Ann. Mo. Bot. Gard.

[5] Oyedemi S.O., Bradley G., Afolayan A.J. (2010). *In vivo* and *in vitro* antioxidant activities of aqueous stem bark extract of *Strychnos henningsii* (Gilg). Afr. J. Pharm. Pharmacol.

[6] Shai L.J., McGwa L.J., Aderogba M.A., Mdee L.K., Eloff J.N. (2008). Four pentacyclic triterpenoids with antifungal and antibacterial activity from *Curtisia dentata* (Burm.f.) C.A. Sm. Leaves. J. Ethnopharmacol.

[7] Pujol J. (2000). Nature Africa: The herbalist Handbook: African Flora, Medicinal Plants.

[8] Dold A.P., Cocks M.L. (2001). Traditional veterinary medicine in the Alice district of the Eastern Cape Province South Africa. S. Afr. J. Sci.

[9] Afolayan A.J., Mbaebie B.O. (2010). Ethnobotanical study of medicinal plants used as anti-obesity remedies in Nkonkobe Municipality of South Africa. Pharmacog. J.

[10] McGaw L.J., Jager A.K., van Staden J. (2000). Antibacterial, anthelmintic and anti-amoebic activity in South African medicinal plants. J. Ethnopharmacol.

[11] Shai L.J., McGaw L.J., Eloff J.N. (2009). Extracts of the leaves and twigs of the threatened tree *Curtisia dentata* (Cornaceae) are more active against *Candida albicans* and other microorganisms than the stem bark extract. S. Afr. J. Bot.

[12] Doughari J.H., Ndakidemi P.A., Human I.S., Benade S. Antimicrobial Susceptibility Profile and Effect of Stem Bark Extracts of *Curtisia dentata* on Multi-drug Resistant Verotoxic *Escherichia coli* and *Acinetobacter spp.* Isolates Obtained from Water and Waste Water Samples.

[13] Yokozawa T., Oura H., Hattori M., Iwano M., Dohi K., Sakanaka S., Kim M. (1993). Inhibitory effect of tannin in green tea on the proliferation of mesangial cells. Nephron.

[14] Dharmananda S. Golinuts and the Uses of Tannins in Chinese Medicine.

[15] Neumann U.P., Berg T., Baha M., Puhl G., Guckelbeger O., Langreh J.M., Neuhaus P. (2004). Long-term outcome of liver transplant for hepatitis C: A 10 year follow-up. Transplantation.

[16] Hodek P., Trefil P., Stiborova M. (2000). Flavonoids—Potent and versatile biologically active compounds interacting with cytochrome P450. Chem. Biol. Int.

[17] Cos P., de Bruyne T., Hermans N., Apers S., Berghe D.V., Vlietinck A.J. (2004). Proanthocyanidins in health care: Current and new trends. Curr. Med. Chem.

[18] Fergusion L.R. (2001). Role of plant polyphenols in genomic stability. Mutat. Res.

[19] Oyedemi S.O., Afolayan A.J. (2011). Antibacterial and antioxidant activities of hydroalcoholic stem bark extract of *Schotia latifolia* Jacq. Asian Pac. J. Trop. Med.

[20] Tural S., Koca I. (2008). Physico-chemical and antioxidant properties of cornelian cherry fruits (*Cornus mas* L.) grown in Turkey. Sci. Hortic.

[21] Wang M., Li J., Rangarajan M., Shao Y., La Voie E.J., Huang T., Ho C. (1998). Antioxidative phenolic compounds from sage (*Salvia officinalis*). J. Agric. Food Chem.

[22] Shami P.J., Moore J.O., Gockerman J.P., Hathorn J.W., Misukonis M.A., Weinberg J.B. (1995). Nitric oxide modulation of the growth and differentiation of freshly isolated acute non-lymphocytic leukemia cells. Leuk. Res.

[23] Dharmananda R. Traces of chloramphenicol in Chinese bee products: Origin, development and resolution.

[24] Rios J.L., Recio M.C. (2005). Medicinal plants and antimicrobial activity. J. Ethnopharmacol.

[25] Richardson J.F., Reith S. (1993). Characterization of a strain of methicillin-resistant Staphylococcus aureus (EMRSA-15) by conventional and molecular methods. J. Hosp. Infect.

[26] Cox R.A., Conquest C., Mallaghan C., Marples R.R. (1995). A major outbreak of methicillin-resistant Staphylococcus aureus caused by a new phage-type (EMRSA-16). J. Hosp. Infect.

[27] Dulger B., Gonuz A. (2004). Antimicrobial activity of some Turkish medicinal plants. J. Biol. Sci.

[28] Highfield E.S., Kemper K.J. (1999). Longwood Herbal Task Force.

[29] Moshi M.J., Mbwambo Z.H., Nondo R.S.O., Masimba P.J., Kamuhabwa A., Kapingu M.C., Thomas P., Richard M. (2006). Evaluation of ethnomedical claims and brine shrimp toxicity of some plants used in Tanzania as traditional medicines. Afr. J. Tradit. Complement. Altern. Med.

[30] Kumar S., Kumar V., Chandrashekhar M.S. (2011). Cytotoxic activity of isolated fractions from methanolic extract of Asystasia dalzelliana leaves by brine shrimp lethality bioassay. Int. J. Pharm. Pharm. Sci.

[31] Kaatz G.W., Seo S.M., Ruble C.A. (1993). Efflux-mediated fluoroquinolone resistance in *Staphylococcus aureus*. Antimicrob. Agents Chemother.

[32] Clinical Laboratory Standards Institute (CLSI) (1994). Performance for Antimicrobial Susceptibility Testing; Standard M100-S5.

[33] Zovko C., Koncic M., Kremwer D., Gruz J., Strnad M., Bisevac G., Kosalec I., Šamec D., Piljac-Žegarac J., Karlović K. (2010). Antioxidant and antimicrobial properties of *Moltkia petrea* (Tratt.) Griseb. Flower, leaf and stem infusions. Food Chem. Toxicol.

[34] Association of Official Analytical Chemists (AOAC) (1990). Official Methods of Analysis.

[35] Harborne J.B. (2005). Phytochemical Methods—A Guide to Modern Techniques of Plant Analysis.

[36] Obadoni B.O., Ochuko P.O. (2001). Phytochemical studies and comparative efficacy of the extracts of some haemostatic plants in Edo and Delta States of Nigeria. Glob. J. Pure Appl. Sci.

[37] Yen G., Chen H. (1995). Antioxidant activity of various tea extract in relation to their antimutagenicity. J. Agric. Food Chem.

[38] Liyana-Pathiranan C.M., Shahidi F. (2005). Antioxidant activity of commercial soft and hard wheat (*Triticum aestivum* L.) as affected by gastric pH conditions. J. Agric. Food Chem.

[39] Re R., Pellegrini N., Proteggente A., Pannala A., Yang M., Rice-Evans C. (1999). Antioxidant activity applying an improved ABTS radical cation decolorization assay. Free Radic. Biol. Med.

[40] Ebrahimzadeh M.A., Pourmorad F., Hafezi S. (2008). Antioxidant activities of Iranian corn silk. Turk. J. Biol.

